# Migration Behavior of Aldehydes in Laminated and Non-Laminated Food-Contact Paper Materials

**DOI:** 10.3390/molecules31213907

**Published:** 2026-10-29

**Authors:** Sijia Sun, Jiaqi Shao, Zeyue Gao, Qianqian Liu, Ying Xu, Li Mu, Gang Li

**Affiliations:** 1 College of Food Science and Engineering, Changchun University, Changchun 130022, China; 13657524519@163.com (S.S.);; 2 Jilin Province Product Quality Supervision and Inspection Institute, Changchun 130023, China

**Keywords:** paper-based food contact materials, HS-GC-MS, food simulants, migration test, barrier effect

## Abstract

Headspace gas chromatography–mass spectrometry (HS-GC-MS) was adopted to detect and screen volatile substances in selected commercially available paper tableware with and without lamination. At an equilibrium temperature of 150 °C and an equilibrium time of 35 min, a total of 25 compounds were detected in non-laminated paper, and 55 compounds were detected in laminated paper. Six volatile compounds were detected in both paper samples with and without lamination, namely *n*-hexanal, furfural, benzaldehyde, *n*-octanal, nonanal, and decanal. The total concentrations of the six target substances in non-laminated paper samples ranged from 254 to 436 μg/mL, while those in laminated paper samples were in the range of 217–1550 μg/mL. The detection rates of all six target analytes (*n*-hexanal, furfural, benzaldehyde, *n*-octanal, nonanal and decanal) in the samples were ≥92.9%. In addition, a systematic study was carried out on the migration patterns of six target substances from laminated and non-laminated paper-based food-contact tableware at varying migration temperatures and migration times in four food simulants (4% acetic acid, 10% ethanol, 50% ethanol and isooctane), and the barrier property of the laminated coating was validated.

## 1. Introduction

As an indispensable and critical part of food safety, the safety of food-contact materials (FCMs) has drawn unprecedented global attention from consumers, producers and regulatory agencies. Throughout their full-cycle contact with food during processing, storage, transportation and consumption, chemicals present in these materials may migrate into the food chain, which may not only impair the sensory properties of food but also introduce chemical hazards and consequently exert a direct impact on public health and well-being [1]. Among various FCMs, paper-based materials have witnessed a booming growth in application and market share for food-packaging purposes. Benefiting from their biodegradability from renewable resources, excellent processability and perceived eco-friendly reputation, such materials are extensively applied to baking paper, paper cups, paper bags, hamburger containers, liquid-food packaging boxes and many other products [2]. Nevertheless, contrary to their seemingly plain appearance, modern paper manufacturing constitutes a complex process with multiple chemicals involved, such as pulping–bleaching agents (chlorine, peroxides), sizing agents (alkyl ketene dimer (AKD), rosin), coating materials (polyethylene (PE), polylactic acid (PLA), fluorinated oil-barrier additives), and printing inks [3]. Residual monomers, additives, degradation products and impurities from these processing aids are major sources of Non-Intentionally Added Substances (NIASs). Migration of these substances into food under given conditions such as temperature, contact time and food characteristics represents one of the greatest challenges in the field of FCM safety [4,5]. Laminated–coated paper is a composite material prepared by coating plastic pellets onto the paper surface via an extrusion–lamination process. Polyethylene (PE) and polypropylene (PP) pellets are commonly used for lamination. Owing to numerous advantages such as light weight, low cost and favorable barrier properties, it has been widely applied in the manufacture of food packaging [6]. Non-laminated paper is manufactured by applying an eco-friendly plastic-free coating onto the paper substrate. The coating features high barrier performance, reduced water adhesion on the paper surface and strong hydrophobicity. Inter-coating adhesion can be achieved at a given temperature, which allows the material to serve as an alternative to laminated paper or plastic films for single-use packaging. This material is biodegradable and recyclable through direct repulping, and its fibers can be reused. In theory, a continuous, uniform laminated layer functions as an effective physical barrier against substance migration from paper-based food-contact tableware to food [7,8]. Laminated–coated paper and non-laminated paper share similar cellulose fiber types (mainly virgin softwood/hardwood chemical pulp for food-grade cupstock) but differ greatly in composition, coating, thickness, manufacturing procedure, additives and application scenarios. Non-laminated paper is pure paper substrate without polymer coating; its main component is cellulose, with minor hemicellulose and lignin. It relies on papermaking additives, including wet-strength agents, retention aids and fillers to maintain strength, and its typical paper thickness ranges from 0.20 to 0.30 mm [9]. In contrast, laminated–coated paper consists of the same paper base plus an extruded low-density polyethylene (LDPE) layer, with coating grammage generally 15–20 g/m^2^ (ca.15–20 μm thickness) on the food-contact surface. The total wall thickness of laminated–coated paper is slightly higher than that of non-laminated paper [10]. During manufacturing, laminated–coated paper undergoes extrusion coating at 280–320 °C before cup forming, while non-laminated paper directly goes through printing, die-cutting and forming without thermal extrusion lamination. In addition to papermaking additives, laminated–coated materials contain processing aids for LDPE, such as antioxidants and slip agents. Due to the hydrophobic PE barrier, laminated–coated paper cups are widely used for hot/cold aqueous beverages. Non-laminated paper lacks liquid barrier capacity and easily absorbs water, so it is mostly applied for dry food packaging rather than long-time liquid holding [11]. Previous studies have demonstrated that the laminated coating can remarkably inhibit the migration of plasticizers (phthalates) and mineral oils [12,13,14,15]. However, the barrier effect of laminated coatings has not yet been systematically validated with a large sample set. Key unresolved issues include whether such barrier performance is universal, whether the coating provides consistent migration-inhibiting effects toward analytes differing in molecular weight, polarity, volatility and other physicochemical properties, and whether micro-defects generated during lamination (uneven thickness, pinholes, incomplete edge coverage) could lead to partial or total loss of barrier function [16,17,18].

Nowadays, the presence and migration of volatile compounds in food-contact materials have drawn widespread concern among researchers worldwide. Previous investigations have confirmed numerous volatile analytes in paper-based food-contact tableware with highly complex sources [19,20]. Some volatile compounds are derived from the plant-fiber raw materials. Lignin, hemicellulose and cellulose may undergo pyrolysis or degradation during pulping, bleaching and high-temperature treatment, generating common degradation products including furfural, benzaldehyde and a range of low-carbon aldehydes [21,22]. Various functional additives added during production and their decomposition products, as well as contaminants such as printing inks and adhesives introduced from recycled fibers, constitute additional sources of volatile substances in paper-based food-contact tableware [23,24]. Of all the determined aldehydes, *n*-hexanal exhibited a markedly elevated average concentration in printed paperboard compared with unprinted base paper. *N*-hexanal has been reported as an oxidation marker of linoleic acid in previous investigations [25,26]. Previous studies have characterized the migration behaviors of the aforementioned aldehydes at elevated temperatures. However, the majority of available works are limited to individual simulants or single compounds. No systematic investigation has been carried out on the parallel migration rules of multiple substances in complex food simulant systems [27]. From a chemical perspective, aldehydes, including *n*-hexanal, furfural, benzaldehyde, nonanal, decanal and *n*-octanal, are typical markers for the oxidative degradation of unsaturated fatty acids and can reflect the oxidation degree of lipids. Meanwhile, furfural indicates the thermal degradation of lignocellulose. As electrophilic substances, aldehydes can form DNA/protein adducts and trigger oxidative stress at high concentrations [28]. Furfural poses the highest health concern, showing genotoxicity, hepatorenal toxicity and carcinogenic potential in animal assays [29,30]. Benzaldehyde is generally safe at dietary exposure; despite positive in vitro chromosomal aberration findings, it is rapidly metabolized to benzoic acid in vivo [31]. The saturated aliphatic aldehydes (hexanal, *n*-octanal, nonanal, decanal) exhibit low acute toxicity and rapid conversion to carboxylic acids according to Joint FAO/WHO Expert Committee on Food Additives (JECFA) evaluations, mainly causing mucosal irritation without convincing carcinogenic or genotoxic evidence at food-relevant doses [32]. Their reported in vitro cytotoxicity occurs at unrealistically high levels. In current migration investigations of food-contact materials and articles, only a restricted range of target migrants are detected in most cases, and existing research mainly concerns well-characterized components, including conventional additives and monomers. Non-Intentionally Added Substances (NIASs), trace-level degradation products and unknown migrants in complex matrices are poorly covered. Quantification of merely a few regulated compounds fails to reflect the full migration profile of materials under practical-use conditions. Most migration tests adopt only a single food simulant, conventional temperature–humidity conditions and short-term immersion. Real-life exposure scenarios, such as pH, ionic strength and lipid components of practical food matrices, as well as long-term aging and repeated-use cycles, are not fully taken into account, which results in limitations in the characterization of migration behaviors. Díaz-Galiano, F.J. [33] subjected polypropylene microwave containers to a 10-day test in 10% ethanol at 40 °C, which systematically clarified the special migration behaviors and inhibition mechanisms of highly hydrophobic volatile compounds in high-temperature oil–water mixed systems.

In the present work, headspace gas chromatography–mass spectrometry (HS-GC-MS) was used for the detection and screening of volatile compounds in commercial paper tableware with and without lamination. Common volatile constituents across samples were selected, followed by migration experiments (4% acetic acid, 10% ethanol, 50% ethanol and isooctane) on representative substances detected in laminated and non-laminated papers. Most existing literature has focused solely on the study of individual aldehydes or fatty aldehydes derived from lipids. This research aims to systematically explore their migration behaviors in various food simulants and validate the barrier effect of the laminated coating.

## 2. Results and Discussion

### 2.1. Identification of Migration Targets

**Figure 1:**
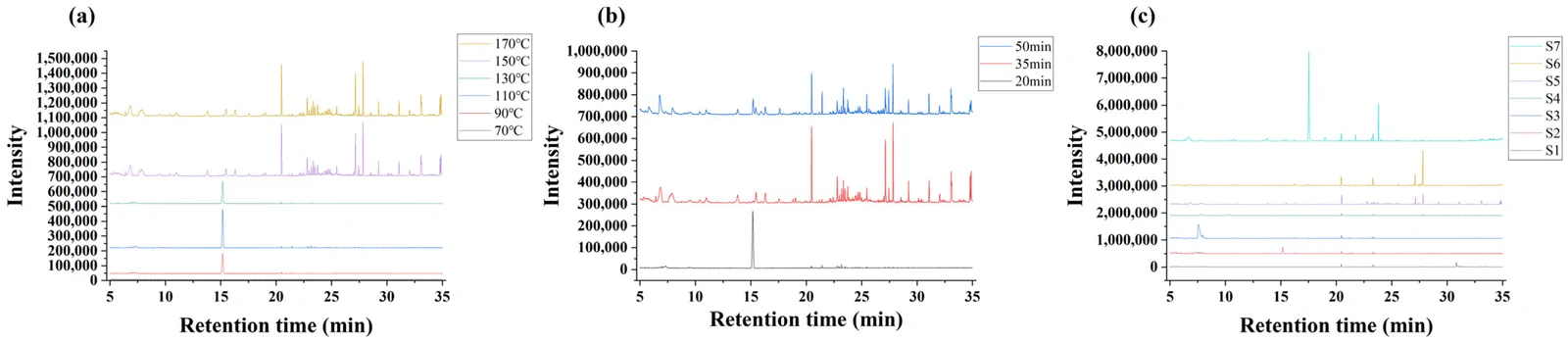
(**a**) Equilibrium temperature; (**b**) equilibration time; and (**c**) total ion current chromatogram for volatile compounds in paper-based food-contact tableware.

Equilibrium temperature is a critical parameter affecting the sensitivity of headspace analysis. At excessively low temperatures, volatile substances cannot be sufficiently released from the sample matrix. In contrast, excessively high temperatures may lead to the pyrolysis of the sample matrix or degradation of target analytes, producing impurity peaks that interfere with the analysis. Equilibration time determines the degree to which volatile components reach partition equilibrium between the sample and the headspace gas phase. An insufficient equilibration time leads to incomplete partition equilibrium and unstable response values. Although a longer equilibration time can improve sensitivity, it will reduce analytical efficiency. To comprehensively capture volatile components from paper tableware with and without a lamination-coating layer, the samples prepared in Section 3.2.1 were analyzed in SCAN mode using headspace gas chromatography–mass spectrometry (HS-GC-MS). The headspace sampling conditions were optimized. The response intensities of target volatile compounds were compared under six equilibrium temperatures (70, 90, 110, 130, 150, 170 °C) and three equilibration times (20, 35, 50 min). An equilibrium condition of 150 °C for 35 min was finally selected as the optimum parameter (Figure 1a,b). Under this condition, volatile compounds can be efficiently released and enriched without inducing thermal decomposition of the sample.

Volatile substances in samples S1–S3 were screened using the optimized equilibrium temperature and equilibration time, and the results are shown in Figure 1c and Table 1. A total of 25 (Table A1) compounds were detected in non-laminated paper products. Among these 25 detected compounds, aldehydes, including nonanal, decanal, *n*-hexanal, furfural, benzaldehyde and *n*-octanal, were all identified, with their concentrations ranging from 0.01 to 300.78 μg/mL. Benzaldehyde, a common aromatic aldehyde [34], was detected in all samples, and its concentration ranged from 4.84 to 58.74 μg/mL. Benzaldehyde originates from natural plant-derived components or additives introduced during processing. The concentration of nonanal ranged from 25.41 to 48.58 μg/mL, while that of *n*-hexanal varied between 30.39 and 66.04 μg/mL. The identified aldehyde compounds are derived from thermal degradation or oxidation processes of cellulose and hemicellulose present in paper fibers [35,36,37]. In addition, *n*-butyl acrylate, 1,4-dioxene and several fluorine-containing compounds were also detected. These substances are generally associated with industrial additives such as adhesives, water-repellent agents and printing inks used during paper manufacturing [38]. The detection results indicate that non-laminated paper products contain non-natural additives introduced during production. The screening results revealed the diversity of volatile substances in non-laminated paper-based food-contact tableware. These volatile compounds originate not only from degradation products generated by the material itself but also from exogenous compounds introduced during processing. This finding suggests that non-laminated paper-based food-contact tableware may pose a migration risk during use.

After screening for volatile substances in laminated paper-based food-contact tableware samples (S4–S7), a total of 55 (Table A2) compounds were detected. Aldehydes were ubiquitous and present at relatively high concentrations. Among them, decanal exhibited concentrations ranging from 0.00 to 1053.99 μg/mL in multiple samples, with the highest value of 1053.99 μg/mL observed in sample S6. Nonanal, *n*-hexanal and benzaldehyde were also widely detected, and benzaldehyde reached its maximum concentration of 371.54 μg/mL in sample S7. Such aldehydes may be derived from thermo-oxidative degradation of the lamination polymer or heat-induced decomposition of cellulose and hemicellulose within the paper-based substrate [39,40]. In addition, 2-ethylhexanol, 2-ethylhexyl acetate, and *n*-butyl acrylate, as well as various alkanes and alkenes, were detected. These compounds are generally related to plasticizers, solvents or residual monomers used in the processing of the laminated layer [41,42], indicating that lamination treatment introduces volatile compounds associated with conventional plastic products. Overall, laminated paper-based food-contact tableware released a more complex profile of volatile substances with higher concentrations, suggesting that it poses a greater migration risk during use.

A comparative analysis of volatile organic compounds (VOCs) from laminated and non-laminated paper-based food-contact tableware samples was carried out. Six volatile substances were commonly detected in these samples, and the results are shown in Table 2. These shared compounds are primarily derived from lipid oxidation-degradation, thermochemical breakdown of wood-derived fibers, and residual chemical additives introduced during paper manufacturing [43,44,45]. Specifically, *n*-hexanal, benzaldehyde, *n*-octanal, nonanal and decanal were the predominant aldehydes among the shared compounds, serving as typical markers for the oxidative degradation of unsaturated fatty acids. The presence of furfural directly indicates the degradation of wood-fiber raw materials under heating conditions. High detection rates were obtained for the six target compounds in seven samples. *n*-hexanal, furfural, benzaldehyde, nonanal and decanal were found in 100% of samples; the detection rate of *n*-octanal was 92.9%. Accordingly, these analytes are suitable for further migration-pattern research. Considering the subsequent migration tests on real samples (S1–S7), six substances, including *n*-hexanal, furfural, benzaldehyde, *n*-octanal, nonanal and decanal, were chosen as research targets.

### 2.2. Migration Test

#### 2.2.1. Comparison of Migration Between Laminated and Non-Laminated Paper

According to the method described in Section 3.2.9 and combined with the migration-rate calculation formula Equation (1) in Section 3.2.8, the migration behaviors of target analytes from seven kinds of paper-based food-contact tableware in aqueous food, acidic food, alcoholic food and fatty food were investigated. The migration-rate results of the samples are shown in Table 3.

Results from migration experiments revealed remarkable differences in the migration behaviors of seven paper-based food-contact tableware samples among the four food simulants. The 4% acetic-acid food simulant exhibited the strongest stimulating effect on the migration of target analytes. The migration rates of samples S1, S2 and S3 ranged from 12.77% to 15.85%, while the migration rate of sample S4 was 9.11%. Isooctane ranked second, with migration rates between 0.33% and 3.02%, and the maximum value of 3.03% was observed in sample S4. Compared with the migration rates obtained in the other three food simulants, 50% ethanol induced migration, with values ranging from 0.61% to 2.61%. In contrast, the migration rates in 10% ethanol varied from 0 to 3.49%. A comparison of migration results among the seven samples showed that the migration rates of the six target analytes were slightly higher in non-laminated samples than in laminated samples. In 4% acetic-acid simulant, the migration rates ranged from 10.01% to 15.85% for the non-laminated group and from 0.46% to 9.11% for the laminated group; the non-laminated group exhibited generally higher migration rates than the laminated group. In the isooctane simulant, sample S4 from the laminated group achieved the maximum migration rate of 3.02%, followed by sample S2 from the non-laminated group at 1.30%, and no obvious inter-group difference was observed. All samples presented relatively low migration rates in both 50% ethanol and 10% ethanol simulants. In summary, the laminated layer exerted a certain inhibitory effect on migration in some food simulants, while the overall migration rates remained at a low level.

#### 2.2.2. Migration Temperature

##### Migration Behaviors of *n*-Hexanal, Furfural and Benzaldehyde in 4% Acetic Acid Simulant

**Figure 2:**
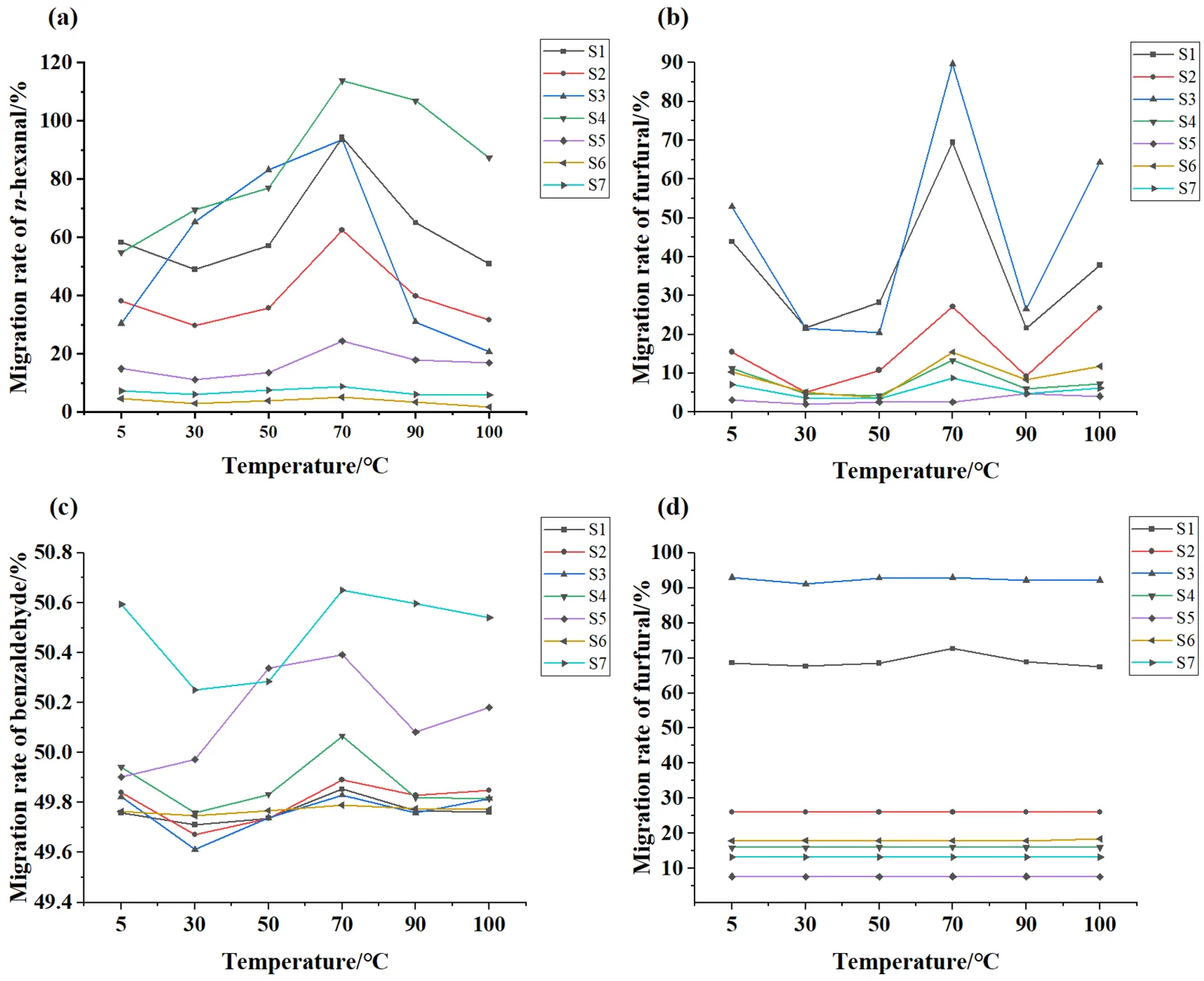
Migration behavior of (**a**) *n*-hexanal, (**b**) furfural, (**c**) benzaldehyde in 4% acetic-acid simulant, and (**d**) furfural in 10% ethanol simulant.

The food simulant mixtures prepared in Section 3.2.5 were treated according to the method described in Section 3.2.9, and the migration rates of target analytes were calculated using Equation (1) from Section 3.2.8. Under the experimental conditions, only three compounds, namely *n*-hexanal, furfural and benzaldehyde, were detected among the six screened-target substances, while the remaining three target analytes were not found. The migration behaviors of the three target substances in 4% acetic acid simulant are illustrated in Figure 2.

As shown in Figure 2a, *n*-hexanal exhibited strong temperature sensitivity, and its migration rate exceeded 100% at 70 °C. At 5 °C, the migration rates ranged from 4.65% to 58.33%. The migration rates of non-laminated samples (S1, S2, S3) were generally higher than those of laminated samples S5, S6 and S7. When the temperature increased to 70 °C, the migration rates reached their maximum values. The migration rates of non-laminated samples S1, S2 and S3 rose to 94.42%, 62.53% and 93.53%; laminated sample S8 exhibited an extremely high migration rate of 113.86%. As the temperature further increased to 90 °C and 100 °C, the migration rates of all samples decreased from the peak values obtained at 70 °C. It is noteworthy that the migration rate of *n*-hexanal from laminated sample S4 exceeded 100% at 70 °C and 90 °C. This phenomenon can be attributed to the conversion of certain precursor substances in the paper-based substrate under the combined effect of the acidic medium (4% acetic-acid simulant) and specific migration temperature [46]. Although the laminated layer inhibited migration in some samples, such an inhibitory effect was inconsistent.

Figure 2b reveals that furfural migration rates varied between 3.07% and 52.88% at 5 °C, with non-laminated samples showing generally higher values than laminated ones. As the migration temperature rose to 30 °C, furfural migration rates decreased for both laminated and non-laminated groups. When the migration temperature rose to 70 °C, the migration rate of furfural reached a peak value. The furfural migration rates of non-laminated samples S1, S2 and S3 increased to 69.52%, 27.09% and 89.72%, respectively. The furfural migration rates of laminated samples ranged from 2.53% to 15.48%. Although an upward trend was also observed, these values were lower than those of non-laminated samples at the same temperature. According to the data analysis, the furfural migration rates of non-laminated samples were generally higher than those of laminated samples at all migration-temperature points, indicating that the laminated layer exerted a certain inhibitory effect on furfural migration.

As shown in Figure 2c, benzaldehyde exhibited extremely stable migration behavior. At 5 °C, the migration rates of non-laminated samples (S1, S2, S3) and laminated samples (S4, S5, S6, S7) were between 49.8% and 50.6%. With the increase in migration temperature, the migration rates of the target analyte in all samples remained in the range of 49.6–50.7%. This result indicated that the migration of benzaldehyde rapidly reached dynamic equilibrium within 2 h. Subsequent temperature elevation had no promoting or inhibitory effect on its migration from the paper-based substrate into 4% acetic-acid simulant. Furthermore, the presence or absence of the laminated layer exerted no observable influence on its migration rate.

##### Migration Behavior of Furfural in 10% Ethanol Simulant

Under the experimental conditions, only furfural was detected among the six target analytes, and the other five target substances were not found. The detection results are shown in Figure 2d.

Obvious migration of furfural occurred at 5 °C. With the increase in temperature, the migration rates of furfural for all samples fluctuated slightly. This indicated that short-term migration was barely affected by temperature in this aqueous simulant, and the migration process reached equilibrium rapidly. Notably, laminated sample S5 showed an abnormally low migration rate of 7.7% at all temperatures, independent of temperature changes. This could be attributed to its unique material composition or lamination technology. The coating layer of S5 was continuous, dense and high-quality, with very few defects, including pinholes and crevices. It acted as an effective physical barrier and greatly prevented migratable substances in the paper substrate from permeating into the 10% ethanol simulant. In contrast, the non-laminated sample S3 exhibited an abnormally high migration rate of 92%, which could be explained by the poor film-forming integrity of its coating layer, accompanied by defects such as micropores and partial insufficient coating. The ethanol component in 10% ethanol simulant could induce swelling of the coating-layer polymers, further enlarging the internal pores of the coating. As a result, the food simulant was able to come into direct contact with the underlying paper-fiber substrate. Additives, impurities and other substances inside the paper were released in large quantities, finally leading to an extremely high migration rate. This sample presented a relatively high migration risk, which should be given priority attention during quality-control procedures.

##### Migration Behavior of *n*-Hexanal, Furfural, Benzaldehyde, *n*-Octanal, Nonanal and Decanal in 50% Ethanol Simulant

**Figure 3:**
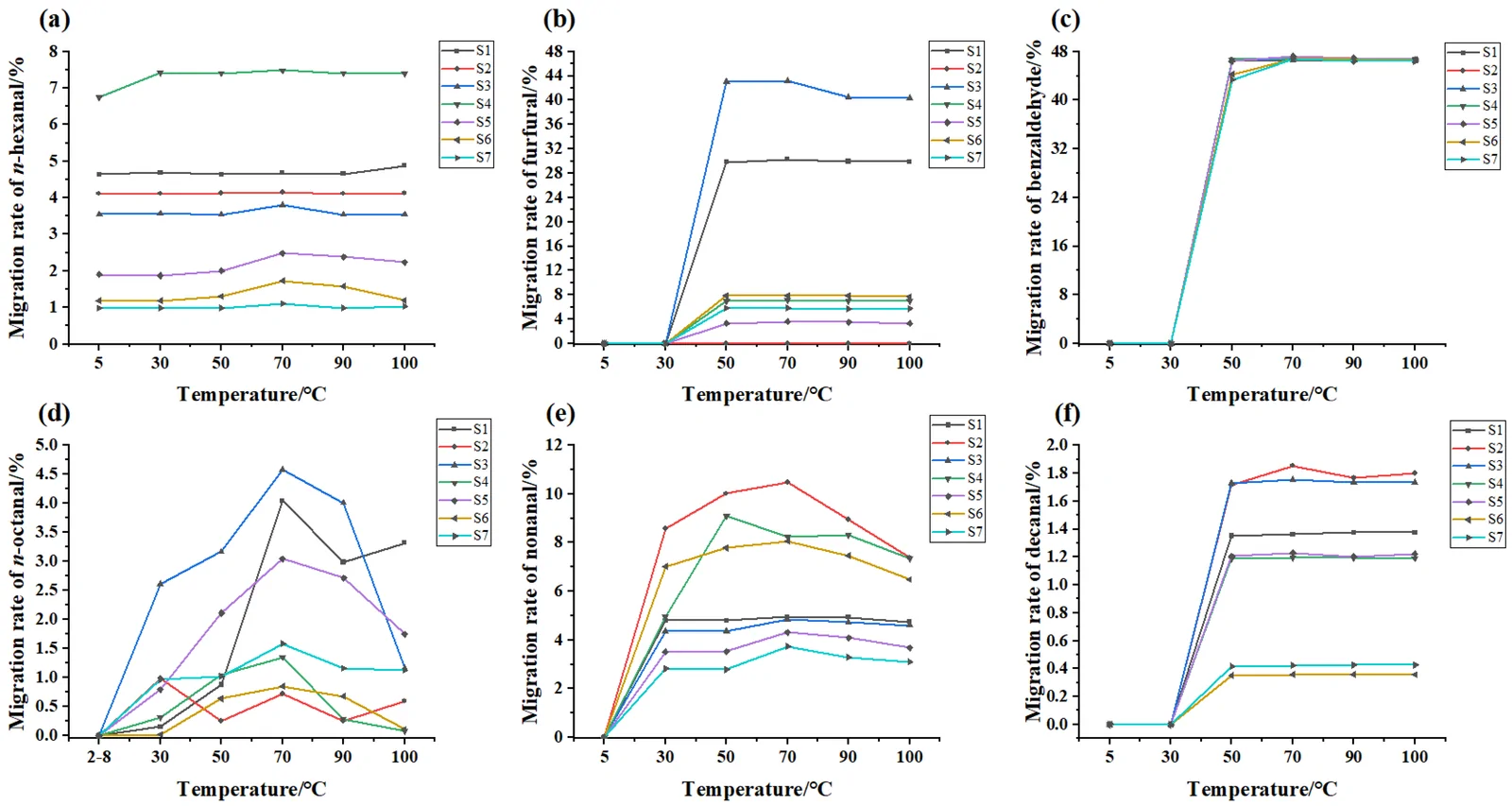
Migration behavior of (**a**) *n*-hexanal, (**b**) furfural, (**c**) benzaldehyde, (**d**) *n*-octanal, (**e**) nonanal, and (**f**) decanal in 50% ethanol simulant.

*N*-hexanal, furfural, benzaldehyde, *n*-octanal, nonanal and decanal were detected under experimental conditions. The results are presented in Figure 3.

As shown in Figure 3a, migration of *n*-hexanal occurred at all tested temperatures (5 °C to 100 °C). At 5 °C, the migration rates of *n*-hexanal ranged from 0.98% to 6.76%. With the increase in temperature, the migration rates generally reached a peak at 70 °C. As the migration temperature further rose, the migration rates of *n*-hexanal in both laminated and non-laminated samples decreased to some extent. The inhibitory effect of the laminated layer was inconsistent. Figure 3b indicates that no furfural was detected at temperatures lower than 30 °C. From 50 °C onward, furfural migration occurred in all samples except S2, which maintained a migration rate of 0% throughout the temperature range. The furfural migration rate of laminated sample S5 was relatively low, ranging from 3.33% to 3.60%, which was lower than that of the other laminated samples in the same group. During the temperature increase from 50 °C to 100 °C, the changes in furfural migration rate for each sample were not. Figure 3c reveals a clear temperature threshold for benzaldehyde migration in 50% ethanol simulant. Benzaldehyde was not detected (0% migration rate) in all samples below 30 °C. Migration initiated at 50 °C with rapid attainment of migration rates. The values fluctuated within a narrow band of 46.4–47.3% at 70 °C, 90 °C and 100 °C. In general, once migration occurred above 50 °C, further temperature rise and the laminated coating exerted no influence on benzaldehyde migration rate. Figure 3d shows that *n*-octanal was undetectable at 5 °C. Migration began at 30 °C (0.014–2.62%). Migration rates increased with temperature and peaked at 70 °C. A general decline in migration rate was observed above 70 °C. For nonanal (Figure 3e), the migration rate of all samples was 0% at 5 °C. Migration initiated at 30 °C, and the migration rate of non-laminated sample S2 was higher than those of the other samples. In the temperature range of 50–70 °C, the migration rates of most samples increased with rising temperature and reached a peak at 70 °C. Figure 3f demonstrates that decanal exhibited migration behavior similar to benzaldehyde. Decanal began to migrate at 50 °C, and its migration rate stayed relatively stable with minor fluctuations over 50–100 °C. The laminated group showed marginally lower migration rates than the non-laminated group.

In 50% ethanol simulant, furfural, benzaldehyde, *n*-octanal, nonanal and decanal began to migrate above 30 °C and were undetectable below this temperature, whereas *n*-hexanal had already migrated at 5 °C. The migration of *n*-hexanal, nonanal and *n*-octanal exhibited temperature-dependent sensitivity, and their migration rates reached a peak at approximately 70 °C. By contrast, after reaching the threshold migration temperature, the migration rates of benzaldehyde, furfural and decanal did not change with the continuous increase in temperature. For *n*-octanal, the migration rates of laminated samples (S4, S5, S6, S7) were lower than those of non-laminated samples (S1, S3). Nevertheless, the inhibitory effect of the laminated layer was not obvious for *n*-hexanal and nonanal, and considerable differences existed among individual samples. Compared with the vigorous migration in 4% acetic acid, target analytes showed comparatively low migration rates in 50% ethanol. In particular, *n*-octanal displayed a full migration pattern exclusively in 50% ethanol simulant. It indicates that high ethanol simulant possesses a unique extraction effect on certain substances, which is important for evaluating the safety of food-contact materials for alcoholic food.

##### Migration Behavior of *n*-Hexanal, Furfural, Nonanal and Decanal in Isooctane Simulant

**Figure 4:**
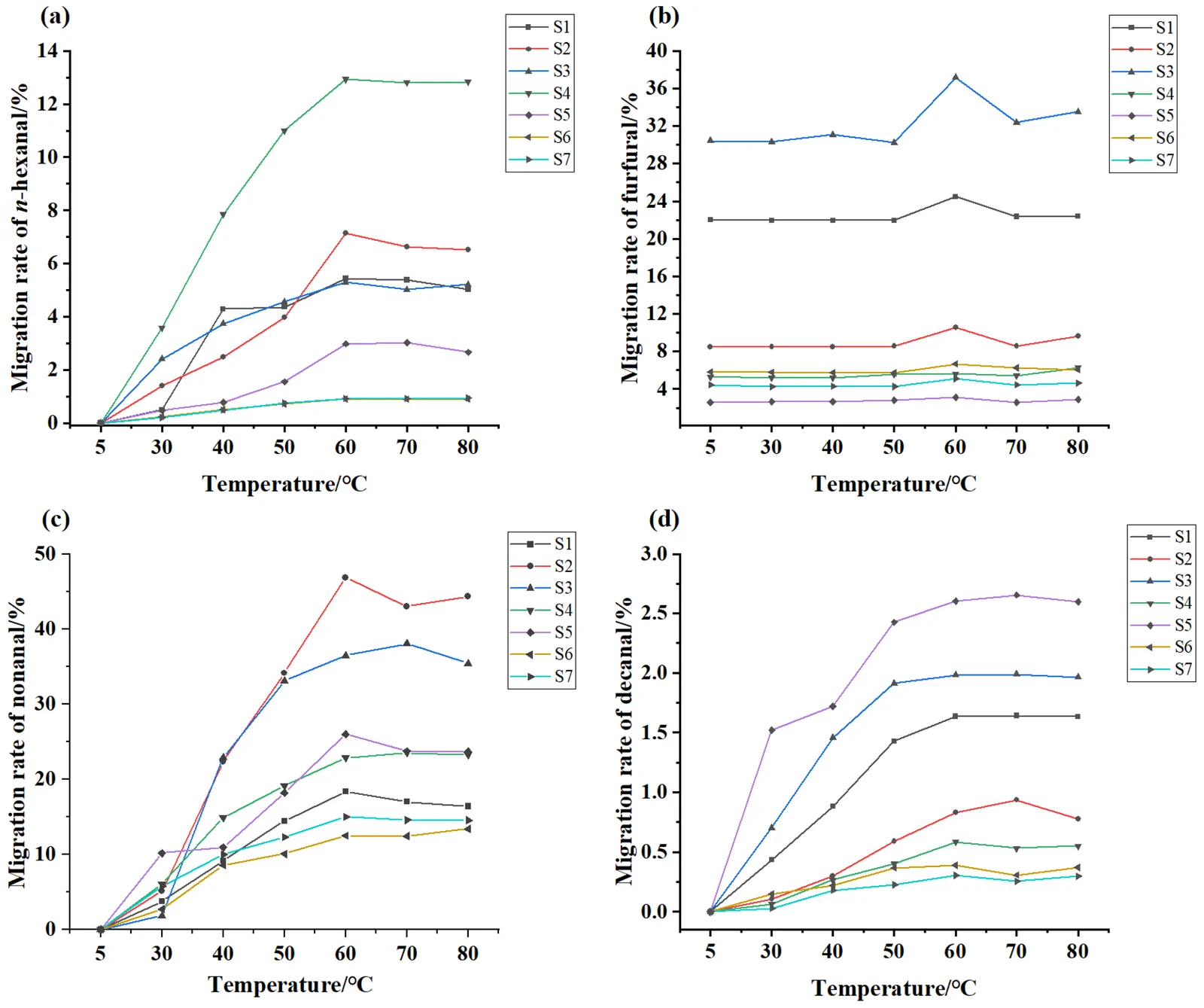
Migration behavior of (**a**) *n*-hexanal, (**b**) furfural, (**c**) nonanal, and (**d**) decanal in isooctane simulant.

*N*-hexanal, furfural, nonanal and decanal were detected among the target substances; the remaining two analytes were absent. The results are presented in Figure 4.

No *n*-hexanal (Figure 4a) migration (0% migration rate) was observed for all samples at 5 °C. Migration started at 30 °C with a remarkable rising trend as temperature increased and reached a plateau or peak at 60 °C. Laminated sample S4 showed high migration rates of 3.59–12.93%, while S6 and S7 had much lower values (0.25–0.92%). The barrier-inhibition performance of the coating varied greatly among samples. Furfural (Figure 4b) showed detectable migration in isooctane at 5 °C. At this low-temperature condition, non-laminated samples (S1–S3) yielded migration rates generally exceeding the laminated samples (S4–S7). Migration rates for both groups stayed relatively stable with minor fluctuations when the temperature rose from 30 °C to 80 °C. As shown in Figure 4c, nonanal was not detected at 5 °C. Migration initiated at 30 °C and exhibited a strong positive correlation with temperature, with the migration rate reaching its maximum at approximately 60 °C. The migration rate increase of nonanal in non-laminated samples S2 and S3 was particularly drastic. The migration rate of S2 soared from 5.14% at 30 °C to 46.83% at 60 °C. The migration rates of laminated samples (S4, S5, S6, S7) were generally lower than those of non-laminated samples (S1, S2, S3) at all tested temperatures. Decanal (Figure 4d) was not detected at 5 °C. Migration initiated at 30 °C, and the migration rate increased continuously with rising temperature, reaching a peak in the range of 60–70 °C.

The comprehensive migration experimental results from four food simulants (4% acetic acid, 10% ethanol, 50% ethanol and isooctane) demonstrated that the migration behavior of target substances strictly followed the “like-dissolves-like” principle. Among lipophilic compounds, *n*-hexanal and nonanal presented the highest migration rates and the strongest temperature sensitivity in isooctane, with their migration rates generally peaking at 60–70 °C. Under acidic conditions, the migration rate of *n*-hexanal even exceeded 100%. As a critical physical barrier, the laminated layer could inhibit migration in most cases. However, its barrier performance was affected by temperature, simulant properties and the type of migrating substance. A barrier effect was observed for most target analytes under acidic and fatty conditions, while the inhibition efficiency toward *n*-hexanal varied. It was found that measurable migration of *n*-octanal occurred exclusively in 50% ethanol simulant, emphasizing that proper simulant selection is essential for full-scale safety evaluation. Consequently, multiple parameters such as migrant chemistry, food matrix, application temperature and material barrier technology should be integrated into the safety assessment of paper food-contact tableware.

#### 2.2.3. Migration Time

##### Migration Behavior of *n*-Hexanal, Furfural and Benzaldehyde in 4% Acetic Acid Simulant

**Figure 5:**
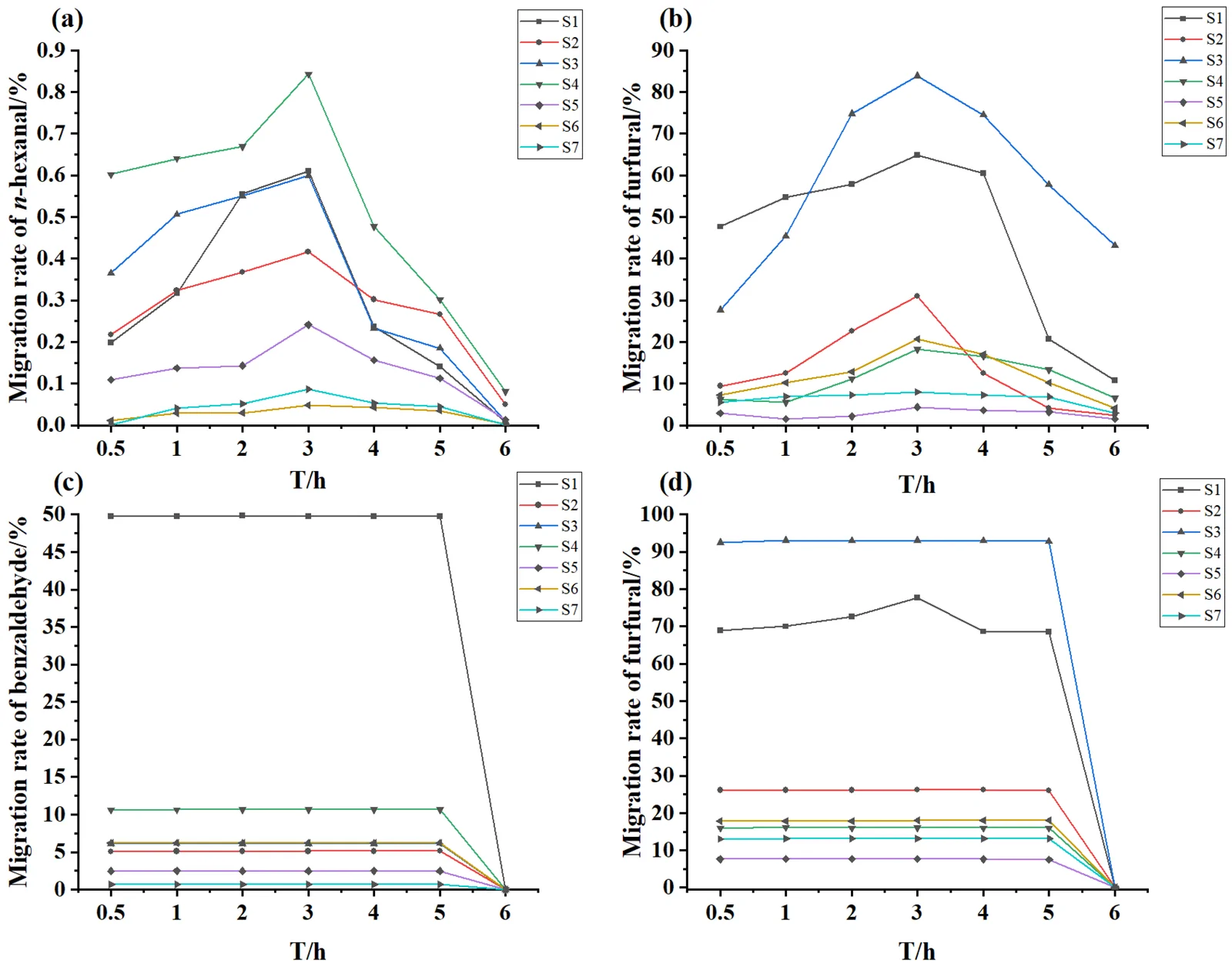
Migration behavior of (**a**) *n*-hexanal, (**b**) furfural, (**c**) benzaldehyde in 4% acetic acid simulant, and (**d**) furfural in 10% ethanol simulant.

Under the tested conditions, only three substances, including *n*-hexanal, furfural and benzaldehyde, were detected, while the remaining three target analytes were not detected. The results are shown in Figure 5.

As shown in Figure 5a, the migration rate of *n*-hexanal remained at an overall very low level (<0.85%) under acidic conditions at 70 °C and presented a gentle trend over time. From 0.5 h to 3 h, the migration rates of most samples increased slowly with the extension of immersion time and reached the maximum value at approximately 3 h. Furfural (Figure 5b) migration rate followed a unimodal pattern: a sharp initial rise followed by a gradual decline. Migration rose quickly over time and commonly peaked at 3 h. Non-laminated samples exhibited higher overall migration rates than laminated samples. As shown in Figure 5c, the migration behavior of benzaldehyde was relatively stable at the initial stage, yet an abrupt change occurred after long-term migration. Migration was detected in all samples at 0.5 h. Migration rates of all samples stayed highly stable from 0.5 h to 5 h, with fluctuations below 0.5%. At 6 h, however, benzaldehyde migration for every sample plummeted to 0%. This suggests that prolonged contact under acidic conditions at 70 °C caused full decomposition or precursor conversion of benzaldehyde, resulting in non-detection.

At 70 °C in 4% acetic-acid simulant, the three substances exhibited distinctly different migration-behavior patterns. Benzaldehyde remained stable at 49.8% in the early stage but was completely degraded at 6 h. The migration rate of furfural decreased after reaching its peak at 3 h. *n*-hexanal showed only minor variation with an overall very low migration rate. The migration of furfural was a rapid kinetic process; its release maximum was achieved within 3 h, followed by a decline caused by near-equilibrium concentration in the simulant, reverse adsorption or substance decomposition. The stability of benzaldehyde was broken after 5 h, leading to its complete transformation. The laminated layer exerted a sustained and effective inhibitory effect on furfural, which possessed a high migration rate. The influence of the laminated coating on *n*-hexanal was inconsistent, while the migration of benzaldehyde was independent of the laminated layer. Compared with temperature-variable experiments conducted at a fixed duration (2 h), the time-dependent experiments at 2 h equivalent conditions more clearly revealed the time points at which each migrant reached migration equilibrium or underwent decomposition. This finding provides important reference value for risk assessment of actual contact durations between paper-based materials and hot acidic food.

##### Migration Behavior of Furfural in 10% Ethanol Simulant

Only furfural was detected among all target compounds, and the remaining five analytes were absent. The results are presented in Figure 5d.

As shown in Figure 5d, migration of furfural was observed in all samples at 0.5 h. For non-laminated samples, S3 exhibited the highest migration rate of 92.46%, followed by S1 (68.88%) and S2 (26.08%). For laminated samples, the migration rates of S4, S6 and S7 were 15.97%, 17.75% and 13.06%, respectively, while S5 showed a relatively low migration rate of 7.65%. When the migration time was extended from 1 h to 5 h, the migration rates of all samples did not fluctuate drastically, indicating excellent stability. The migration rate of non-laminated sample S1 fluctuated within a narrow range of 68.61–77.65%, reaching a maximum value of 77.65% at 3 h. For laminated sample S4, its migration rate remained stable at 15.97–16.19%. Sample S5 maintained a consistently low migration rate of 7.51–7.72%. Over the whole period from 0.5 h to 5 h, the migration rates of the samples did not show a consistent monotonic increasing or decreasing trend. Nevertheless, the furfural migration rates of all samples abruptly dropped to 0% once the migration time reached 6 h. This phenomenon is fully consistent with the behaviors of benzaldehyde in 4% acetic acid at 70 °C and *n*-octanal in 50% ethanol at 70 °C after 6 h migration.

##### Migration Behavior of *n*-Hexanal, Furfural, Benzaldehyde, *n*-Octanal, Nonanal and Decanal in 50% Ethanol Simulant

**Figure 6:**
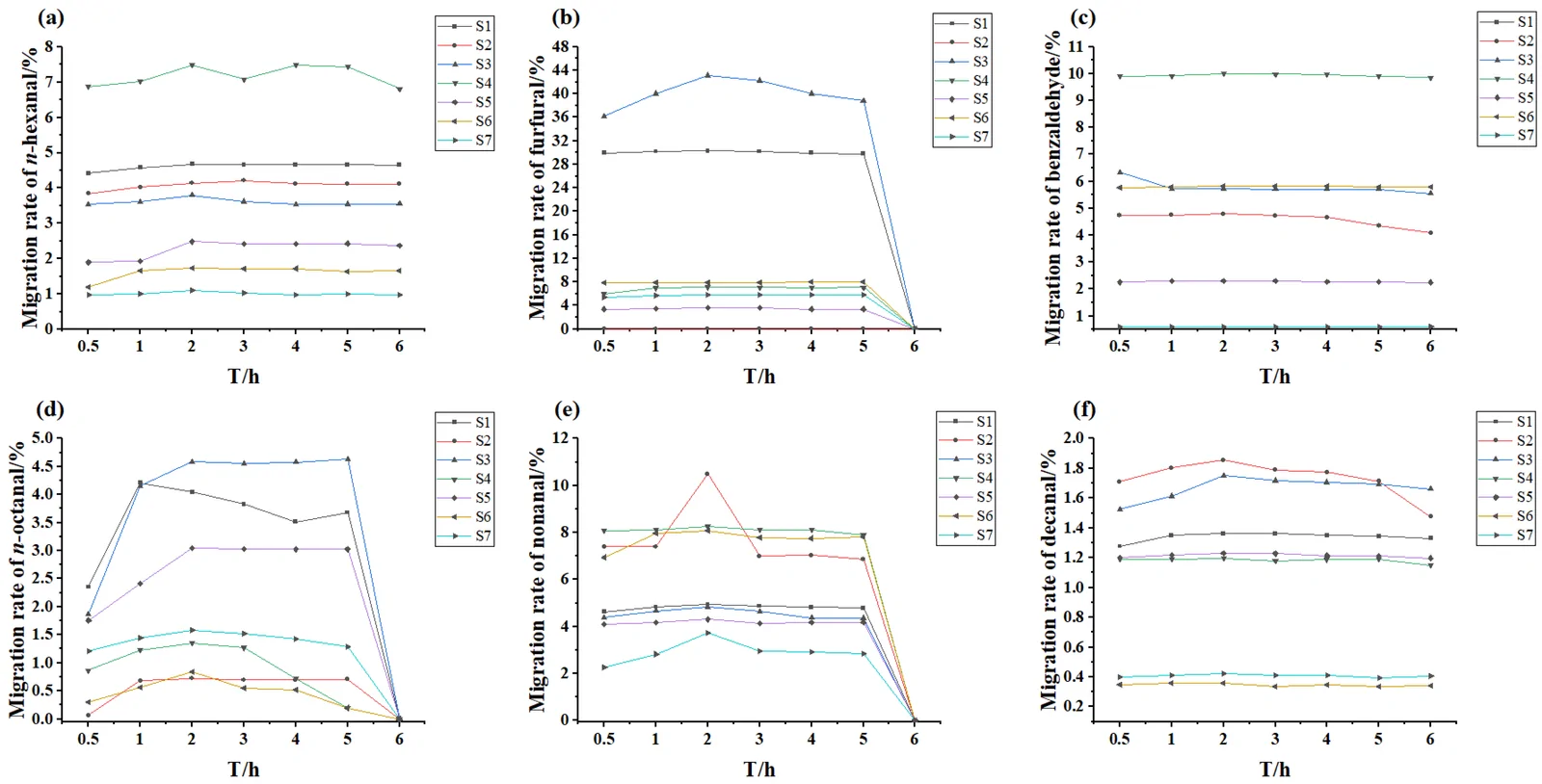
Migration behavior of (**a**) *n*-hexanal, (**b**) furfural, (**c**) benzaldehyde, (**d**) *n*-octanal, (**e**) nonanal, and (**f**) decanal in 50% ethanol simulant.

*n*-Hexanal, furfural, benzaldehyde, *n*-octanal, nonanal and decanal were detected under experimental conditions. The results are presented in Figure 6.

As shown in Figure 6a, the migration rate of *n*-hexanal exhibited a gentle unimodal change over time. The migration rates of all samples increased gradually from 0.5 h to 2 h, reached their maximum values within 2–3 h, and then decreased slowly and tended to stabilize after 3 h. The inhibitory effect of the laminated coating varied among individual samples. Figure 6b indicates that furfural migration rose initially, remained stable, and then underwent a sudden shift. Samples S1, S3, S4, S6 and S7 showed rising migration rates from 0.5 h to 2 h and peaked near 2 h. Sample S2 displayed anomalous results with 0% migration at every time point. Migration values fluctuated around the peak from 2 h to 5 h, before an abrupt fall to 0% for all samples at 6 h. Figure 6c reveals that benzaldehyde achieved a high migration rate at 0.5 h, which stayed nearly constant thereafter. Sample S1 showed distinctive sustained high-migration behavior, while the laminated layer exerted stable inhibition on the remaining samples. It was shown that the migration rate of *n*-octanal increased rapidly from 0.5 h to 2 h, peaked at 2 h, and then decreased slowly during 2–5 h in Figure 6d. Non-laminated samples S1 and S3 presented relatively high migration rates, with peak values of 4.05% and 4.58%, respectively. The migration rates of laminated samples S4, S6 and S7 were low, ranging from 0.85% to 1.59%. When the migration time reached 6 h, the migration rate of *n*-octanal in all samples dropped abruptly to 0%. The migration rate of nonanal showed a trend of rapid increase followed by a decrease over time (Figure 6e). The migration rate rose sharply from 0.5 h to 2 h and reached its peak at 2 h, after which it began to decline. Overall, the migration rates of laminated samples were lower than that of non-laminated sample S2 but close to those of non-laminated samples S1 and S3. When the migration time reached 6 h, the migration rate of nonanal in all samples dropped abruptly to 0%, which was consistent with the migration behavior of furfural. Figure 6f reveals that decanal shared a similar migration profile with benzaldehyde and maintained good stability over time. Decanal never showed zero-migration at any sampling time.

At 70 °C in 50% ethanol simulant, the migration rates of benzaldehyde and decanal remained relatively stable from 0.5 h to 6 h without dropping to zero. This indicated that these compounds were chemically stable under such conditions and reached migration equilibrium rapidly. The migration rates of furfural, nonanal and *n*-octanal peaked at approximately 2 h, followed by variation or decline, and all decreased to zero at 6 h. This phenomenon suggests that these substances were unstable upon prolonged exposure to high-temperature ethanol, with a critical point for chemical decomposition or transformation. The migration rate of *n*-hexanal fluctuated gently over time and reached its peak between 2 h and 3 h, while no zero-migration was observed at 6 h. The laminated coating exerted an inhibitory effect on the migration of furfural, benzaldehyde, *n*-octanal, nonanal and decanal, except for sample S4, which presented a relatively high migration rate. Its inhibition toward *n*-hexanal was inconsistent. This experiment reveals that at a fixed temperature, migration risk depends not only on the migration amount but also on the duration of chemical stability of migrants in food simulants.

##### Migration Behavior of *n*-Hexanal, Furfural, Nonanal and Decanal in Isooctane Simulant

**Figure 7:**
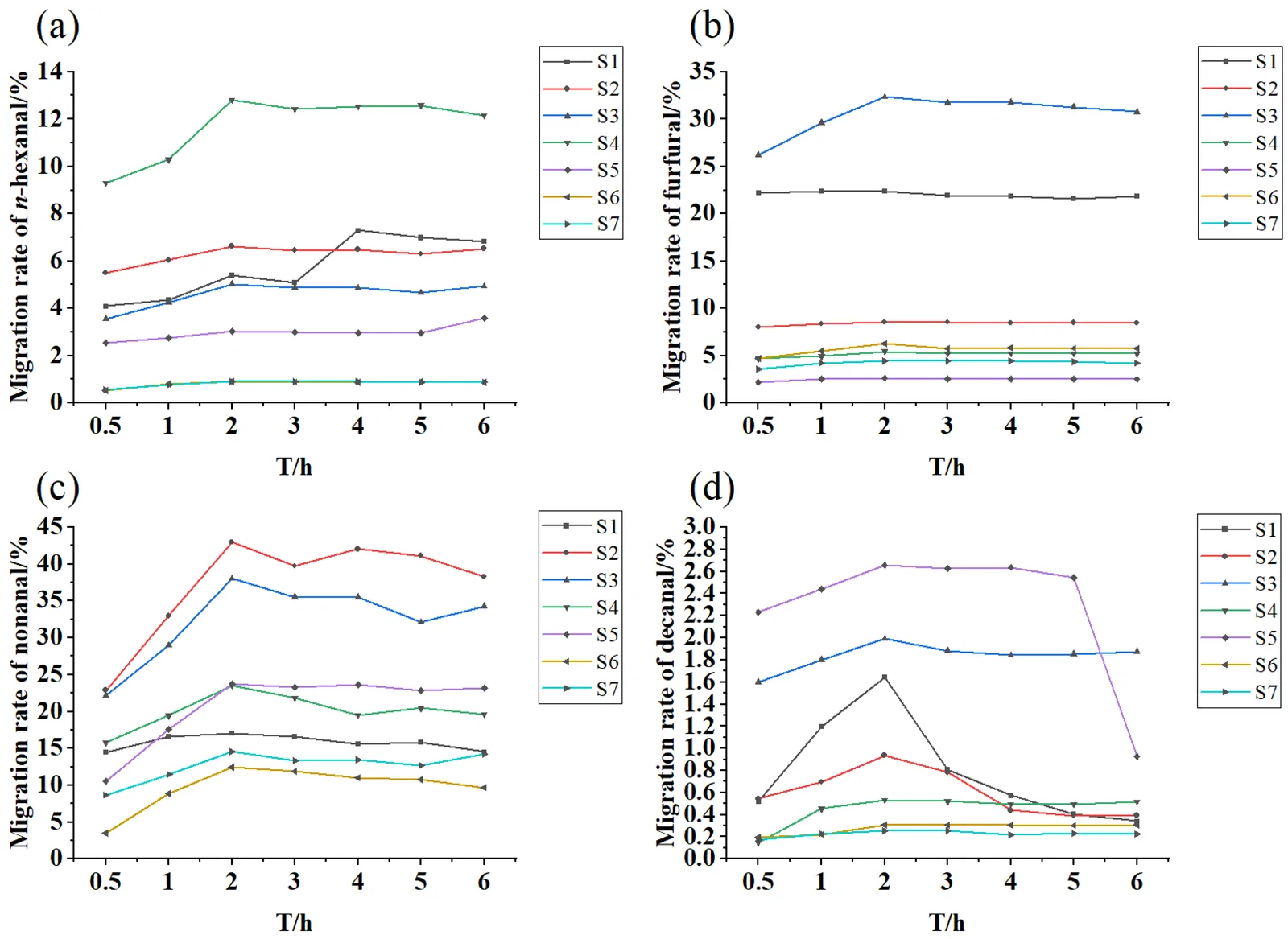
Migration behavior of (**a**) *n*-hexanal, (**b**) furfural, (**c**) nonanal, and (**d**) decanal in isooctane simulant.

Under the tested conditions, four substances, including *n*-hexanal, furfural, nonanal and decanal, were detected, while the remaining two target analytes were not detected. The results are shown in Figure 7.

As shown in Figure 7a–d, the migration rate of *n*-hexanal presented an increasing trend with prolonged immersion time. The migration rates of all samples rose rapidly as time increased from 1 h to 2 h and reached a peak-plateau stage within 2–4 h. During 4–6 h, the migration rates fluctuated slightly at a high level without any decline. The peak migration rate of laminated sample S4 was higher than those of non-laminated samples, whereas the other laminated samples (S5, S6, S7) exhibited relatively low migration rates, indicating that the effect of the laminated coating varied among samples. Furfural reached migration equilibrium rapidly and remained stable thereafter. Relatively high migration rates were detected at 0.5 h. Non-laminated samples ranged 7.99–26.23%, generally higher than laminated samples (3.60–4.70%). Migration values stayed constant without obvious fluctuation from 1 h to 6 h. It suggests that furfural attained dynamic equilibrium in isooctane within 1 h, and further time extension exerted no influence on migration level. The laminated layer provided persistent inhibition. Nonanal exhibited a migration profile similar to *n*-hexanal, yet with a steeper rising amplitude. Measurable migration rates (3.51–22.89%) were already observed at 0.5 h, among which non-laminated S2 and S3 were higher than the rest. Migration increased rapidly and peaked at 2 h. Laminated samples S4, S6 and S7 maintained lower migration levels than non-laminated samples during the entire test. Decanal’s migration rate rose rapidly and then declined over immersion time. Low-level migration (0.15–2.23%) was observed at 0.5 h. Following the peak at 2 h, all samples exhibited a sustained and remarkable drop in migration rate, with values substantially lower than the peak at 6 h.

At 70 °C in isooctane simulant, furfural reached migration equilibrium rapidly within 1 h, and its migration rate remained stable for a long time, indicating that its transfer from the paper-based material to the fatty simulant was completed quickly. The migration rates of *n*-hexanal, nonanal and decanal increased with immersion time and peaked at approximately 2 h. After reaching the peak, *n*-hexanal and nonanal entered a plateau stage or slow-decay phase, whereas decanal exhibited a continuous decline after 2 h. In isooctane simulant, the laminated coating produced an obvious migration-inhibitory effect on most samples for furfural, nonanal and decanal. However, its inhibitory performance toward *n*-hexanal was inconsistent. Distinctly different from the abrupt “zero-migration” phenomenon observed for several compounds at 6 h in 50% ethanol, all analytes remained detectable throughout 0.5–6 h in isooctane simulant with more stable migration profiles. This confirms that fatty-food environments exert different effects on the chemical stability of these substances. Such a comparison highlights the necessity of assessing migration risks separately according to food type (aqueous, acidic, alcoholic, fatty), since the same compound presents completely distinct stability and migration-kinetics behaviors in different food simulants.

## 3. Materials and Methods

### 3.1. Materials and Reagents

Non-Laminated Paper Samples (Figure 1): Paper cups made from white sugarcane pulp (S1), paper bowls prepared from yellow straw pulp (S2), paper bowls made from yellow wheat-straw pulp (S3) purchased from a local supermarket.

**Figure 8:**
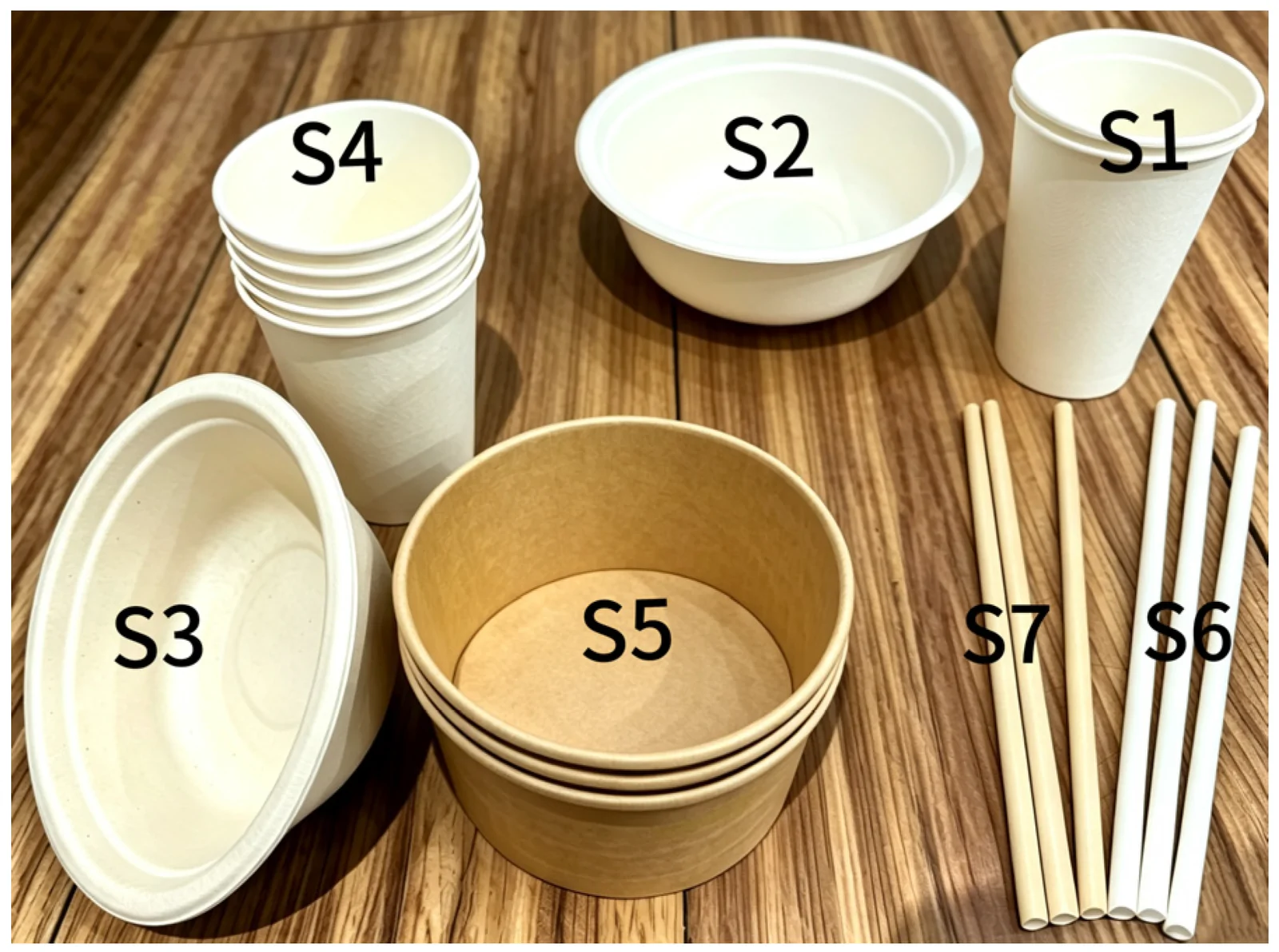
Test samples (S1–S7).

Laminated Paper Samples (Figure 8): Paper cups (S4), yellow food containers (S5), white straws (S6) and yellow straws (S7) were obtained from a local supermarket.

Regents: Glacial acetic acid, 95% ethanol, isooctane, furfural (≥99.5%), *n*-hexanal (99%), acrylate (≥99.5%), nonanal (95%), decanal (≥98%), benzaldehyde (99.5%), *n*-octanal (99%), and acetophenone (99.5%) were obtained from Ron-Reagent (China). White vinegar and edible oil were obtained from a local supermarket.

### 3.2. Experimental Methods

#### 3.2.1. Sample Preparation I

Each of the practical samples, S1–S7, was cut into 0.5 cm × 0.5 cm fragments with scissors. A sample of 1.7 ± 0.1 g was transferred into a 20 mL headspace vial. The vial was purged with nitrogen for 30 s and sealed before analysis. Three replicates were performed for every sample.

#### 3.2.2. Preparation of Food Simulants

In accordance with GB 31604.1-2023 National Food Safety Standard [47], general rules for migration tests of food-contact materials and articles and EU Regulation No 10/2011, four food simulants were prepared: 4% (v/v) acetic acid, 10% (v/v) ethanol, 50% (v/v) ethanol and isooctane (analytical-reagent grade).

First, 4% (v/v) acetic acid: A total of 20 mL glacial acetic acid was accurately transferred and diluted with 480 mL deionized water, followed by thorough mixing.

Second, 10% (v/v) ethanol: An aliquot of 50 mL absolute ethanol was diluted with 450 mL deionized water, followed by thorough mixing.

Third, 50% (v/v) ethanol: An aliquot of 250 mL absolute ethanol was diluted with 250 mL deionized water, followed by thorough mixing.

#### 3.2.3. Pretreatment of Actual Samples

Each real sample (S1–S7) was cut into 0.5 cm × 0.5 cm fragments using scissors. Pretreatment was carried out following the GB 5009.156-2016 National Food Safety Standard, general rules for pretreatment of food-contact materials and articles for migration tests. The conventional surface-area-to-volume (S/V) ratio was adopted, where 6 dm^2^ of food-contact material was immersed in 1 L of food simulant.

#### 3.2.4. Sample Preparation II

The pretreated samples prepared according to the method described in Section 3.2.3 were placed into ground-glass-stoppered Erlenmeyer flasks so that the tableware samples were fully immersed in the food simulant. The flasks were heated in a water-bath pot at a preset temperature for a certain period of time. Afterwards, the flasks were taken out and cooled to room temperature. An aliquot of 5 mL solution was transferred into a 20 mL headspace vial, sealed, and then analyzed by the instrument.

#### 3.2.5. Preparation of Food Simulant Mixtures

The samples prepared in Section 3.2.4 were mixed with the food simulants prepared in Section 3.2.2 according to the procedure described in Section 3.2.3 to obtain food simulant mixtures.

#### 3.2.6. Gas Chromatography Conditions

Headspace Conditions: A headspace autosampler (Model HSS 86.50, Beijing Bo-Sai Technology Co., Ltd., Beijing, China) was used. The equilibrium temperature was set at 150 °C, the loop temperature at 110 °C, and the transfer-line temperature at 120 °C. The equilibration time was 35 min, the GC cycle time 60 min, the injection time 0.5 min, and the pressure-equilibration time 0.5 min. The volume of headspace vials was 20 mL.

Gas Chromatography Conditions: Gas chromatographic analysis was performed on a GC-2010 Plus instrument (Shimadzu Corporation, Kyoto, Japan). A DB-5MS capillary column (30 m × 0.25 mm) was adopted. High-purity helium (purity > 99.999%) was used as the carrier gas with a constant flow rate of 1.0 mL/min. The injector temperature was set at 250 °C, and the splitless injection mode was applied. Temperature ramp protocol: initial temperature was set to 35 °C and held for 2 min; then the temperature was increased at a rate of 3 °C/min to 70 °C and held for 2 min; subsequently, the temperature was increased at a rate of 8 °C/min to 180 °C and held for 2 min; finally, the temperature was increased at a rate of 10 °C/min to 280 °C and held for 2 min.

#### 3.2.7. Mass Spectrometry Conditions

GC-MS analysis was carried out using an Agilent 6890-5973N instrument (Agilent Technologies, Santa Clara, CA, USA). Electron ionization (EI) was adopted. The ion-source temperature was 250 °C, and the electron energy was 70 eV. The full-scan mode was used with a mass-to-charge (*m*/*z*) scanning range of 35–400.

The NIST.17 spectral library was used for matching, and SPSS 27.0 statistical software was employed to analyze the experimental data.

#### 3.2.8. Migration Rate (MR)


(1) $$MR=\frac{C_{t}}{C_{0}}\times 100\%$$

In Equation (1), MR represents the percentage of the migrated substance relative to its initial content in the sample. C_t_ represents the migrated amount of the migrant (μg/mL). C_0_ represents the initial content of the migrant in the sample (μg/mL) [48].

#### 3.2.9. Design of Migration Conditions

Temperature Effect Experiment: Under the migration time of 2 h, the migrated amounts of six key substances, including *n*-hexanal, furfural, benzaldehyde, *n*-octanal, nonanal, and decanal, were determined in food simulants at different temperatures. Six temperature levels (5, 30, 50, 70, 90 and 100 °C) were established for the 4% acetic acid, 10% ethanol and 50% ethanol systems. For the isooctane system, another seven temperature conditions were applied: 5, 30, 40, 50, 60, 70 and 80 °C.

Time Effect Experiment: The migrated amounts of six key substances in food simulants were determined at different migration times under a constant migration temperature of 70 °C. Seven migration-time conditions (0.5, 1, 2, 3, 4, 5 and 6 h) were set for the four food simulant systems.

## 4. Conclusions

Headspace gas chromatography–mass spectrometry (HS-GC-MS) was applied for the comparative analysis of seven commercially available laminated and non-laminated paper-based food-contact materials. At an equilibrium temperature of 150 °C and an equilibrium time of 35 min, a total of 25 compounds were detected in non-laminated paper samples, while 55 compounds were identified in laminated paper samples. Six substances, namely *n*-hexanal, furfural, benzaldehyde, *n*-octanal, nonanal and decanal, were detected with a detection rate ≥92.9% in all seven samples. In accordance with GB 31604.1-2023 National Food Safety Standard for Migration Tests of Food-Contact Materials and Articles, a systematic study was carried out on the migration patterns of the six target analytes from laminated and non-laminated paper-based tableware under different migration temperatures and immersion times in four food simulants (4% acetic acid, 10% ethanol, 50% ethanol and isooctane). Under 4% acetic acid simulant, the total migration rates of target analytes ranged from 10.01% to 15.85% for the three non-laminated paper samples and from 0.46% to 9.11% for the four laminated paper samples. Under 10% ethanol simulant, the total migration rates of target analytes were 0.51–3.49% for non-laminated samples and 0–2.63% for laminated samples. Under 50% ethanol simulant, the total migration rates ranged from 1.89% to 2.61% for the three non-laminated samples and from 0.61% to 1.64% for the four laminated samples. Under isooctane simulant, the total migration rates of target analytes were 0.33–1.30% for non-laminated samples and 0.43–3.02% for laminated samples. Migration patterns of migrants complied well with the like-dissolves-like rule. For lipophilic substances, *n*-hexanal and nonanal achieved their maximum migration rates in isooctane and showed the strongest temperature dependence. As a critical physical barrier, the laminated coating can inhibit migration in most cases. Nevertheless, its barrier efficiency is affected by temperature, simulant properties and the properties of individual migrants.

This study not only investigated the migration mechanism of laminated–coated paper as a food-contact material but, more importantly, systematically clarified for the first time the special migration behavior and inhibition mechanism of strongly hydrophobic volatile substances under high-temperature conditions and oil–water mixed phases by simulating real-world complex food systems. The results revealed the difference in barrier performance between the laminated layer and plastic-free coating and analyzed the influences of manufacturing defects and medium conditions on substance migration. This work possesses important practical significance for optimizing the high temperature production process of paper-based packaging materials and accurately evaluating their sensory-related safety risks in real-food applications containing oil and acidic components. Furthermore, it provides critical data support and scientific evidence for the further improvement of relevant food safety regulations and standards.

## Figures and Tables

**Table 1:** Retention times, linear regression equations, linear ranges and limits of quantitation for *n*-hexanal, furfural, benzaldehyde, *n*-octanal, nonanal, and decanal.

Compound	Retention Time/min	Regression Equation	r^2^	Linear Range (µg/mL)	LOD (µg/mL)	LOQ (µg/mL)
*n*-Hexanal	7.004	y = 15,673x − 24,380	0.9992	1.0~200.0	0.02	0.8
Furfural	7.610	y = 32,304x − 7260.9	0.9999	0.8~200.0	0.02	0.6
Benzaldehyde	13.600	y = 8255.7x + 15,905	0.9996	0.8~200.0	0.02	0.6
*n*-Octanal	15.951	y = 2574.3x − 2199.7	0.9994	0.3~200.0	0.03	0.1
Nonanal	19.949	y = 868.41x − 1011.2	0.9993	0.3~200.0	0.03	0.1
Decanal	22.825	y = 1193.4x − 473.73	0.9992	0.2~200.0	0.03	0.1

y: peak area; x: mass concentration, μg/mL.

**Table 2:** Concentration ranges of target substances with/without laminated paper.

CAS	Target Substances	Retention Time/min	Concentration Range/(μg/mL)	
			Non-Laminated Paper	Laminated Paper
66-25-1	*n*-Hexanal	6.65–7.05	30.4~66.0	51.5~219.8
98-01-1	Furfural	7.54–8.28	0.0~8.4	7.4~21.6
100-52-7	Benzaldehyde	13.67–13.9	4.8~58.7	19.6~371.5
124-13-0	*n*-Octanal	16.12–16.37	0.0~19.2	0.0~104.2
124-19-6	Nonanal	20.42–20.49	25.4~48.6	39.0~74.1
112-31-2	Decanal	23.28–23.38	138.2~300.8	0.0~1054.0

**Table 3:** Total migration rates of six substances in four food simulants.

Samples	Migration Rate (MR)/%			
	4% Acetic Acid Simulant	10% Ethanol Simulant	50% Ethanol Simulant	Isooctane Simulant
S1	12.774	3.491	1.888	0.327
S2	10.011	0.506	2.608	1.302
S3	15.851	1.518	2.152	0.387
S4	9.106	1.111	1.638	3.020
S5	4.518	-	1.591	0.769
S6	0.459	2.629	0.677	0.426
S7	1.389	0.246	0.607	1.266

## Data Availability

The original contributions presented in the study are included in the article, further inquiries can be directed to the corresponding authors.

## Appendix A

**Table A1:** Qualitative and peak-area-normalized quantitative results for volatile compounds from non-laminated paper.

CAS	Retention Time/min	Substance	Concentration/(μg/mL)		
			S1	S2	S3
543-75-9	5.27–6.17	1,4-Dioxin,2,3-dihydro	10.46	19.89	0.51
100-52-7	13.67–13.9	Benzaldehyde	4.84	47.45	39.36
91-20-3	22.71–22.84	Naphthalene	—	—	—
640-19-7	18.95–19.08	Fluoroacetamide	0.61	—	—
124-19-6	20.42–20.49	Nonanal	37.47	37.81	41.28
98-01-1	7.54–8.28	Furfural	2.41	6.34	1.79
112-31-2	23.28–23.38	Decanal	279.26	213.21	219.57
5441-52-1	30.79–30.93	Cyclohexanol,3,5-dimethyl	1.50	—	—
141-32-2	10.55–10.8	Butyl acrylate	—	—	29.37
124-13-0	16.13–16.39	*n*-Octanal	2.15	7.75	19.00
1768-25-8	16.21–16.3	Cyanic acid isobutyl ester	—	—	25.20
98-86-2	18.93–18.99	Acetophenone	—	—	—
620-20-2	20.86–20.92	m-Chlorbenzyl chloride	—	—	—
6232-21-9	10.54–10.84	2-Cyanoglycine	—	71.58	29.36
66-25-1	6.59–7	*n*-Hexanal	46.31	52.18	60.62
647-42-7	7.46–7.89	3,3,4,4,5,5,6,6,7,7,8,8,8-Tridecafluoro-1-octanol	—	9.53	16.68
762-62-9	17.46–17.53	1-Pentene,4,4-dimethyl	—	—	20.80
334-68-9	34.26–34.29	1-Fluorododecane	—	—	1.55
111-71-7	10.89–11.05	Heptanal	18.21	48.54	33.44
625-33-2	19.91–20.21	3-Penten-2-one	—	—	—
1708-27-6	20.07–20.18	3-methyl-2,3-dihydrofuran	—	—	—
63839-85-0	25.44–25.56	α-Methyl-1H-indene-1-methanol acetate	—	—	—
264-09-5	25.78–25.84	7H-benzocycloheptene	—	—	—
92-52-4	27.18–27.22	Biphenyl	—	—	—
77376-84-2	36.21–36.29	2-Propenenitrile, 3-[(1,1-dimethylethyl)amino]-	—	—	—

**Table A2:** Qualitative and peak-area-normalized quantitative results for volatile compounds from laminated paper.

CAS	Retention Time/min	Substance	Concentration/(μg/mL)			
			S4	S5	S6	S7
20521-42-0	10.02–11.44	2-ethenyl-2-butenal	277.80	—	—	—
106-35-4	10.05–10.33	3-Heptanone	—	—	—	18.74
141-32-2	10.57–10.88	Butyl acrylate	—	—	—	18.92
109-73-9	10.64–10.84	*n*-Butylamine	—	—	—	—
111-71-7	10.87–11.15	Heptanal	—	—	230.48	12.63
1703-52-2	11.06–11.54	2-Ethyl-5-methyl furan	3.29	—	—	—
4208-57-5	11.16–11.36	1-(2-Furyl)butan-1-one	—	25.67	—	—
2941-23-3	11.57–11.80	2-Amino-1-cyclopentene-1-carbonitrile	—	—	—	—
111-37-5	11.96–12.15	n-Ethyl-n′-methylethylenediamine	—	—	—	—
123-05-7	13.26–13.44	2-Ethylhexanal	—	—	—	236.53
50767-53-8	13.29–13.16	β-D-lyxo-Hexopyranose, 1,6:3,4-dianhydro-2-deoxy-	—	—	—	—
100-52-7	13.68–13.86	Benzaldehyde	19.61	129.88	38.79	371.54
5961-33-1	14.74–14.91	3-Methyl-3-phenyl-azetidine	—	—	—	48.86
3777-69-3	15.36–15.56	2-Amylfuran	—	131.45	—	188.27
124-13-0	16.12–16.37	*n*-Octanal	14.49	66.20	104.19	82.42
288-14-2	17.15–17.35	Isoxazole	—	—	264.57	—
104-76-7	17.40–17.62	2-Ethylhexanol	—	—	81.18	—
98-86-2	18.94–19.14	Acetophenone	7.49	—	—	—
75-56-9	19.98–20.08	1,2-Epoxypropane	—	—	—	—
124-19-6	20.45–20.61	Nonanal	41.88	68.06	74.05	65.08
620-20-2	20.87–20.97	m-Chlorbenzyl chloride	1.54	—	—	—
103-11-7	21.68–21.82	2-Ethylhexyl acrylate	—	—	—	68.38
6570-87-2	21.76–21.79	3,4-Dimethyl-1-pentanol	—	—	—	—
87773-62-4	21.9–22.03	1-(3-methylfuran-2-yl)propan-2-one	—	1.41	—	—
13151-04-7	22.1–22.16	1-Heptene, 5-methyl	—	9.24	—	—
91-20-3	22.72–22.81	Naphthalene	—	0.52	—	—
13151-10-5	22.91–22.97	1-Octene, 6-methyl-	—	—	11.42	—
7212-53-5	22.94–23.01	5-Methylheptan-1-ol	—	183.66	—	—
921-47-1	23.12–23.21	2,3,4-Trimethylhexane	—	—	—	24.49
112-31-2	23.29–23.46	Decanal	316.37	—	1053.98	890
4032-93-3	23.46–23.53	2,3,6-Trimethylheptane.	—	112.06	—	—
34246-57-6	23.71–23.80	3-Propan-2-ylbenzaldehyde	86.95	217.58	5.93	—
75-83-2	24.81–24.96	2,2-Dimethylbutane	—	—	—	373.62
69064-36-4	25.64–25.69	Trans-2-Dodecenol	—	—	15.95	—
1137-12-8	27.06–27.15	Longicyclene	—	497.10	99.50	—
92-52-4	27.18–27.26	Biphenyl	—	72.81	—	—
475-20-7	27.75–27.85	(+)-Longifolene	—	—	302.43	—
4630-07-3	27.78–27.87	Valencene	270.08	—	—	—
928-95-0	29.4–29.46	Trans-2-hexen-1-ol	—	—	9.18	—
334-68-9	30.29–30.54	1-Fluorododecane	78.11	—	—	—
79-06-1	30.8–30.9	Acrylamide	—	—	—	3.44
55320-58-6	31.15–31.22	5,5-Dimethylhexanal	—	—	—	4.10
62108-21-8	31.97–32.09	6-Ethyl-2-methyl-decane	—	20.68	—	—
74421-18-4	31.98–32.06	1-(Hexyloxy)-3-methylhexane	—	—	—	7.65
4237-44-9	32.75–32.79	2-(1-Phenylethyl)phenol	—	—	—	—
2801-84-5	33.01–33.06	2,4-Dimethyldecane	—	—	7.09	—
66-25-1	6.65–7.05	*n*-Hexanal	31.8	114.5	180.62	219.75
98-01-1	7.57–8.22	Furfural	10.34	21.57	9.22	12.58
96-04-8	33.02–33.09	2,3-Heptanedione	—	—	—	—
4032-94-4	34.82–34.98	2,4-dimethyloctane	—	673.46	—	—
543-75-9	5.29–5.96	1,4-Dioxin,2,3-dihydro	—	20.05	20.85	—
28292-42-4	5.48–5.94	3-Aminoheptane	—	—	—	6.16
4359-77-7	6.33–6.43	3-Methylidenepentan-2-one	—	—	—	3.18
107-25-5	6.3–6.57	Methoxyethene	—	—	—	—
89763-93-9	6.43–8	2-Fluoro-4-(trifluoromethyl)benzaldehyde	—	—	—	4.98
